# Akuter Winkelblock: Ein ophthalmologischer Notfall in der medizinischen Notaufnahme

**DOI:** 10.1007/s00063-021-00790-8

**Published:** 2021-02-13

**Authors:** S. Nuessle, J. Luebke, D. Boehringer, T. Reinhard, A. Anton

**Affiliations:** 1 Klinik für Augenheilkunde, Medizinische Fakultät, Albert-Ludwigs-Universität Freiburg, Killianstr. 5, 79106 Freiburg, Deutschland; 2 ADMEDICO Augenzentrum, Olten, Schweiz

**Keywords:** Palpation, Diagnostik, Symptom, Notaufnahme, Winkelblockglaukom, Palpation, Diagnostic, Symptom, Emergency room, Angle closure glaucoma

## Abstract

**Hintergrund:**

Die Symptome des akuten Winkelblocks, ein Notfallereignis, das ohne rechtzeitige Therapie zur irreversiblen Erblindung führen kann, sind vielfältig. Diese können initial als internistische oder neurologische Erkrankungen gedeutet werden, wenn Kopfschmerzen, Pupillenstarre oder Übelkeit im Vordergrund stehen. Ziel unserer Studie war es, die Rate belastender und invasiver Diagnostik durch primäre Vorstellung bei Nichtophthalmologen bei akutem primären Winkelblock zu erfassen.

**Methode:**

Retrospektive Single-Center-Studie von Patienten mit akutem primärem Winkelblock. Zur Identifizierung dieser wurden alle Patienten erfasst, bei denen im Universitätsklinikum Freiburg, Klinik für Augenheilkunde im Zeitraum 2014–2018 eine chirurgische Iridektomie (5-133.0) oder Iridotomie durch Laser (5-136.1) durchgeführt wurde. Anschließend erfolgte durch Akteneinsicht die Datenanalyse zur Prüfung der Ein- und Ausschlusskriterien sowie des Krankheitsverlaufs.

**Ergebnisse:**

Eingeschlossen wurden 91 Patienten mit akutem primären Winkelblock. Davon stellten sich 28 % (*n* = 25) initial bei nichtophthalmologischen Fachdisziplinen vor. In dieser Patientengruppe erhielten 56 % (*n* = 11) eine nichtzielführende Diagnostik, wobei bei 32 % (*n* = 8) eine kraniale Bildgebung und bei 8 % (*n* = 2) eine Lumbalpunktion durchgeführt wurde.

**Schlussfolgerung:**

Bei akutem primären Winkelblock zeigt sich eine hohe Rate an nicht wegweisender Diagnostik durch Nichtophthalmologen, weshalb dieses Krankheitsbild fächerübergreifend präsent sein sollte. Bei unspezifischen Symptomen, wie Kopfschmerzen, Übelkeit und Erbrechen sowie Pupillenstarre, muss an die Möglichkeit eines akuten Augeninnendruckanstiegs durch einen akuten Winkelblock gedacht und das frühzeitige Hinzuziehen eines Ophthalmologen erwogen werden.

## Abstract (en)

**Background:**

The symptoms of acute angle closure, an emergency event that can lead to irreversible blindness without timely treatment, are diverse. Initially, these can be interpreted as internal or neurological diseases if headaches, pupil rigidity or nausea are in the foreground. The aim of our study was to assess the rate of harming and invasive diagnostics after primary presentation of patients with acute primary angle closure to nonophthalmologists.

**Methods:**

Retrospective single center study of patients with acute primary angle closure. To analyze these patients, all patients who were treated by surgical iridectomy (5–133.0) or iridotomy by laser (5–136.1) in the period 2014–2018 at the Eye Center at Medical Center, University of Freiburg (Germany), were identified. Subsequently, data analysis was carried out through file inspection to check the inclusion and exclusion criteria and the course of the disease.

**Results:**

In total, 91 patients with acute primary angle closure were included. Of these, 28% (*n* = 25) initially presented to nonophthalmological disciplines. In this patient group 56% (*n* = 11) received nontargeted diagnostics, with cranial imaging in 32% (*n* = 8) and lumbar puncture in 8% (*n* = 2).

**Conclusion:**

Acute primary angle closure is associated with a high rate of nontargeted diagnostics by nonophthalmologists. Therefore, the clinical picture of acute angle closure should be in mind across all disciplines. With unspecific symptoms such as headaches, nausea and vomiting as well as pupil rigidity, the possibility of an acute increase in intraocular pressure caused by acute angle closure must be considered and early consultation with an ophthalmologist is recommended.

Der akute Winkelblock ist ein potenziell zur Erblindung führendes ophthalmologisches Notfallereignis. Eine Erstkonsultation in der medizinischen Notaufnahme ist aufgrund der unspezifischen Symptome, wie Kopfschmerzen, Pupillenstarre oder Übelkeit, denkbar. Diese könnten als internistische oder neurologische Erkrankungen gedeutet werden mit dem Risiko einer nichtzielführenden Diagnostik und verzögerten Therapieeinleitung. In diesem Beitrag werden das Vorstellungsverhalten bei akutem Winkelblock und die durchgeführte Diagnostik außerhalb der Augenheilkunde sowie die Auswirkungen vorgestellt.

Der akute Winkelblock ist ein ophthalmologisches Notfallereignis, das ohne rechtzeitige Therapie zur irreversiblen Erblindung führen kann. Die plötzliche Augeninnendruckerhöhung kann zu einer Minderperfusion und irreversiblen Zerstörung der retinalen Ganglienzellen und ihrer Nervenfasern führen. In der Vergangenheit wurden hierfür häufig auch die Begriffe „Glaukomanfall“ oder „akutes Winkelblockglaukom“ synonym verwendet. Jedoch wird empfohlen, die Bezeichnung „akutes Winkelblockglaukom“ auf Patienten mit daraus resultierender glaukomatöser Optikusneuropathie zu beschränken und bei alleiniger Augeninnendruckerhöhung durch Kammerwinkelblockierung den Begriff „akuter Winkelblock“ zu verwenden [7].

Glaukomerkrankungen sind die zweithäufigste Erblindungsursache weltweit. Geschätzt wird, dass von einem Engwinkelglaukom weltweit insgesamt 15,7 Mio. Menschen betroffen sind, wobei die Prävalenz bei europäischen Patienten bei ca. 0,25–0,6 % liegt, da hier Formen des Offenwinkelglaukoms überwiegen [2, 6, 11]. Zu den Risikofaktoren des akuten primären Winkelblocks zählen eine asiatische Abstammung, eine anatomisch flache Vorderkammer, eine verkürzte Bulbuslänge bei Hyperopie, eine zunehmende Linsendicke, ein höheres Alter und das weibliche Geschlecht [10, 13, 17]. Beim akuten Winkelblock entsteht die exzessive Augeninnendrucksteigerung aufgrund einer Verlegung des Trabekelmaschenwerks, das für den primären Abfluss des Kammerwassers zuständig ist, durch die periphere Iris. Ohne rechtzeitige Drucksenkung kann dies bis zur irreversiblen Erblindung führen. Unterteilt werden primäre und sekundäre Formen sowie Mechanismen mit und ohne Pupillarblock. Beim Pupillarblock, dem häufigsten Mechanismus, entsteht durch Annäherung der zentralen Irisrückfläche an die Linsenvorderfläche eine Widerstandserhöhung des Kammerwasserflusses von der Hinter- in die Vorderkammer. Dies erzeugt einen Druckgradienten, der zu einer Vorwölbung der peripheren Regenbogenhaut und dadurch Verlegung des Trabekelmaschenwerks im iridokornealen Kammerwinkel führt ([13]; Abb. 1). Das Behandlungsprinzip des akuten Winkelblocks zielt darauf ab, zunächst schnellstmöglich den Augeninnendruck zu senken gefolgt von der mechanischen Lösung des Winkelblocks. Die chirurgische Iridektomie und die Iridotomie durch Laser stellt die beiden Therapiemöglichkeiten der Wahl zur Beseitigung der Blockade dar, indem eine Öffnung in der Iris zur zusätzlichen Kammerwasserzirkulation erzeugt wird.

**Figure 1:**
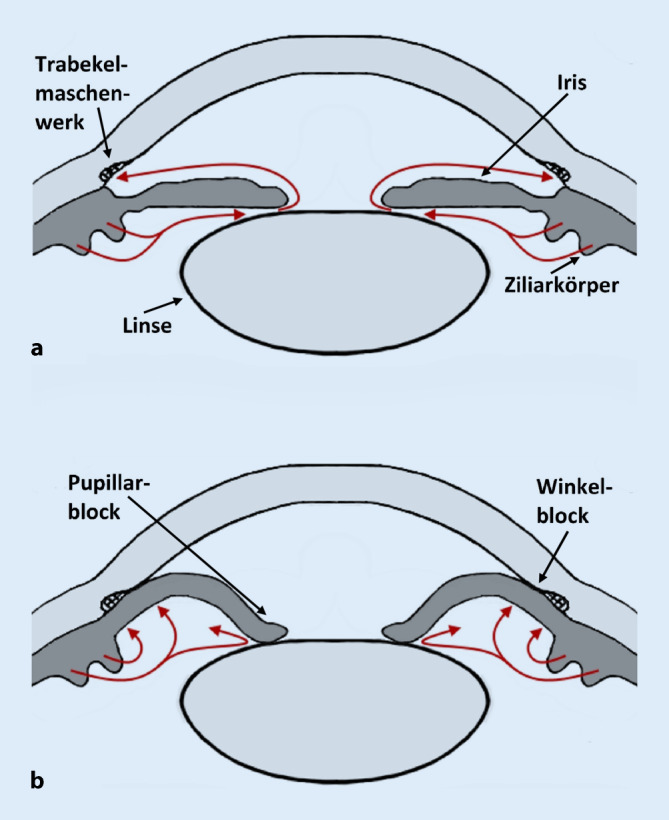

Aufgrund der irreversiblen Nervenfaserschädigung, die bis zur Erblindung führen kann, und der zum Teil ausgeprägten klinischen Symptomatik zählt der akute Winkelblock zu den Notfalldiagnosen in der Augenheilkunde und sollte schnellstmöglich erkannt und therapiert werden. Da die Beschwerden dabei jedoch vielseitig sind und nicht ausschließlich auf die Augen begrenzt sein müssen, können die Symptome initial auch als internistische oder neurologische Erkrankungen fehlgedeutet werden. So kann es zwar zu einer Sehverschlechterung durch ein druckbedingtes Hornhautödem oder Augenschmerzen kommen, jedoch treten auch Symptome wie Übelkeit, Erbrechen, Pupillenstarre oder Kopfschmerzen auf [18]. Aufgrund der unspezifischen Symptome ist eine Erstkonsultation außerhalb der Augenheilkunde denkbar. Wegweisend für die Diagnose ist die deutliche intraokulare Druckerhöhung, wobei die palpatorische Augeninnendruckmessung eine wichtige Untersuchungstechnik zur schnellen Eingrenzung darstellt, indem der Bulbus bei Abblick durch beide Zeigefingern auf dem Oberlid mit rotierender Bewegung palpiert wird (Abb. 2). Die Kenntnis der Beschwerden beim akuten Winkelblock sind daher für das differenzialdiagnostische Vorgehen durch Ärzte insgesamt essenziell, um sowohl nichtwegweisende Untersuchungen zu vermeiden als auch eine gezielte und unverzögerte Weiterleitung zum Augenarzt zu gewährleisten.

**Figure 2:**
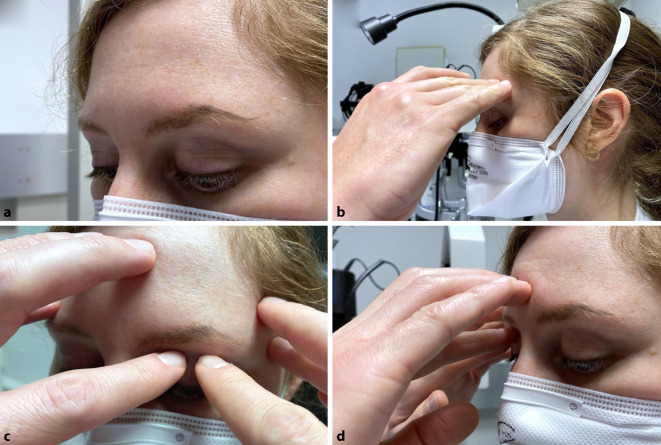

Ziel dieser retrospektiven Studie ist es, die Rate belastender und invasiver Diagnostik durch primäre Vorstellung bei Nichtophthalmologen bei Patienten mit akutem primärem Winkelblock zu erfassen. Unseres Wissens nach gibt es in der Literatur bisher keine Studien, die sich mit dieser Fragestellung befasst haben.

## Methode

### Studiendesign und Population

Bei unserer Analyse handelt es sich um eine retrospektive Single-Center-Studie der Klinik für Augenheilkunde des Universitätsklinikums Freiburgs im Zeitraum vom 01.01.2014 bis zum 31.12.2018. Initial wurde die Datenbank nach der OPS-Kodierung (Operationen- und Prozedurenschlüssel) 5‑133.0 („chirurgische Iridektomie“) oder 5‑136.1 („Iridotomie mit Laser“) durchsucht. Doppelnennungen wegen beidäugiger Behandlung wurden ausgeschlossen. Die verbliebenen Patienten wurden auf die Ein- und Ausschlusskriterien durch Akteneinsicht geprüft. Als Ausschlusskriterien wurden sekundäre Ursachen sowie prophylaktische Eingriffe bei Engwinkelsituation ohne akutes Ereignis definiert. Insgesamt wurden 91 Patienten eingeschlossen, bei denen ein akuter primärer Winkelblock sowie eine ausreichende Dokumentation zur weiteren Datenanalyse vorlagen. Ein positives Ethikvotum der Ethikkommission der Albert-Ludwigs-Universität Freiburg für die Studie liegt vor (Nr. 474/19, am 28.11.2019).

### Datenanalyse und Statistik

Durch Akteneinsicht wurde das Vorstellungsverhalten, die durchgeführte Diagnostik und die dadurch entstehende zeitliche Verzögerung bis zur Diagnose des akuten primären Winkelblocks erfasst. Zudem wurden die Symptome und ophthalmologischen Befunde gesammelt. Nichtdokumentierte anamnestische Angaben oder Befunde wurden als nicht vorhanden gewertet. Als zusätzliche Diagnostik wurde jede diagnostische Maßnahme neben der Augeninnendruckmessung angesehen. Eine dadurch entstehende zeitliche Verzögerung bis zur Diagnosestellung wurde auf Tagesgrenzen gerundet.

Der Zusammenhang zwischen der Häufigkeit der einzelnen Symptome und dem Vorstellungsverhalten wurde mittels χ^2^-Test statistisch untersucht. Ein *p*-Wert < 0,05 wurde als statistisch signifikant angesehen. Auf eine Korrektur für multiples Testen wurde angesichts des explorativen Charakters dieser Untersuchung verzichtet. Zur Berechnung wurde das Statistiksystem R unter Rückgriff auf das Paket „Hmisc“ verwendet [19].

## Ergebnisse

### Patientengruppe

**Figure 3:**
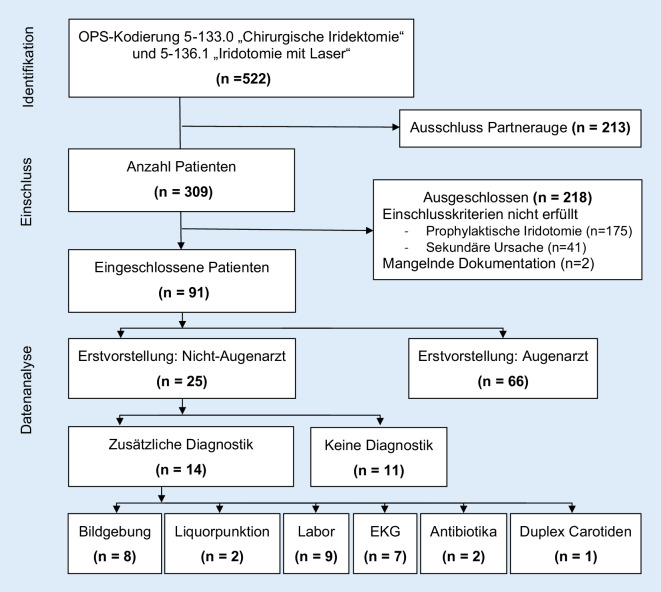

Es konnten 522 Augen, an denen eine Iridektomie bzw. Iridotomie durchgeführt wurde, identifiziert werden. 213 waren Mehrfachnennungen bei beidäugiger Behandlung. Von den 309 identifizierten Patienten wurden 175 Patienten mit prophylaktischer Iridotomie bei Engwinkelsituation, 41 Patienten aufgrund anderer sekundärer Ursache und 2 Patienten aufgrund mangelnder Dokumentation ausgeschlossen. Insgesamt konnten 91 Patienten mit akutem primärem Winkelblock eingeschlossen werden (Abb. 3).

### Vorstellungsverhalten und klinisches Bild

Von diesen 91 Patienten stellten sich 66 (73 %) direkt beim Augenarzt vor, 25 (28 %) konsultierten zunächst einen Nichtophthalmologen. Die primär aufgesuchten nichtophthalmologischen Fachdisziplinen zeigten sich wie folgt: 14 Patienten stellten sich initial in einer Notaufnahme, 5 Patienten bei einem Allgemeinmediziner sowie jeweils ein Patient in einer pädiatrischen Praxis, bei einem Optiker, in einer plastischen Chirurgie (postoperativ nach Blepharoplastik) und in einer Neurochirurgie vor. Zwei Patienten schilderten ihre Symptome während eines bereits bestehenden stationären Aufenthalts anderer Ursache. Bei 2 der 25 Patienten wurde eine zusätzliche Fachdisziplin hinzugezogen (Überweisung aus der Allgemeinmedizin in die Notaufnahme sowie neurologisches Konsil nach stationärer Aufnahme in eine internistische Abteilung).

Detaillierte Aussagen über die häufigsten Symptome und objektiven Befunde sind in Tab. 1 aufgelistet.

**Table 1:** 

Symptom/ Befund	Insgesamt (*n* = 91)	Ophthalmologe (*n* = 66)	Nichtophthalmologe (*n* = 25)	*Nichtophthalmologe* *(+)* *=* *zusätzliche Diagnostik (n* *=* *14)* *(−)* *=* *direkte Überweisung (n* *=* *11)*	
Sehverschlechterung	82 % (*n* = 75)	80 % (*n* = 53)	88 % (*n* = 22)	*(+): 86* *% (n* *=* *12)* *(−): 91* *% (n* *=* *10)*	*p* *=* *0,692*
–	–	***p*** **=** **0,389**		*–*	
Augenschmerzen	47 % (*n* = 43)	42 % (*n* = 28)	60 % (*n* = 15)	*(+): 71* *% (n* *=* *10)* *(−): 45* *% (n* *=* *5)*	*p* *=* *0,188*
–	–	***p*** **=** **0,134**		*–*	
Rötung	23 % (*n* = 21)	15 % (*n* = 10)	44 % (*n* = 11)	*(+): 50* *% (n* *=* *7)* *(−): 36* *% (n* *=* *4)*	*p* *=* *0,495*
–	–	***p*** **=** **0,004**		*–*	
Kopfschmerzen	34 % (*n* = 31)	21 % (*n* = 14)	68 % (*n* = 17)	*(+): 71* *% (n* *=* *10)* *(−): 64* *% (n* *=* *7)*	*p* *=* *0,678*
–	–	***p*** **<** **0,001**		*–*	
Übelkeit/ Erbrechen	24 % (*n* = 22)	17 % (*n* = 11)	44 % (*n* = 11)	*(+): 57* *% (n* *=* *8)* *(−): 27* *% (n* *=* *3)*	*p* *=* *0,135*
–	–	***p*** **=** **0,007**		*–*	
Anisokorie/ Pupillenstarre	14 % (*n* = 13)	0 % (*n* = 0)	52 % (*n* = 13)	*(+): 57* *% (n* *=* *8)* *(−): 45* *% (n* *=* *5)*	*p* *=* *0,561*
–	–	***p*** **<** **0,001**		*–*	

### Diagnostik und Verzögerung

**Figure 4:**
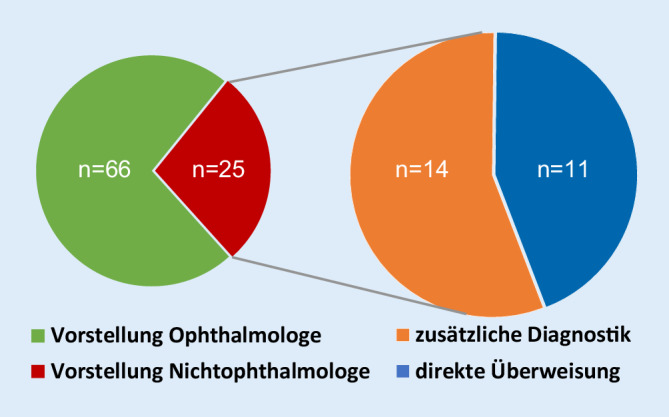

Von den 25 Patienten mit primär nichtophthalmologischer Vorstellung wurden 11 Patienten (44 %) direkt ohne weitere zusätzliche Diagnostik zum Augenarzt überwiesen. Bei den restlichen 14 Patienten (56 %) wurde mindestens eine zusätzliche Diagnostik durchgeführt (Abb. 4). Folgende diagnostische Mittel wurden eingesetzt: 8 Patienten (32 %) erhielten eine kraniale Bildgebung (davon wurde bei 5 Patienten eine alleinige kraniale Computertomographie (cCT), bei 2 Patienten kombiniert mit einer CT-Angiographie und bei einem Patienten eine Magnetresonanztomographie des Schädels (cMRT) durchgeführt), 2 Patienten (8 %) wurden neben der Bildgebung zudem lumbal punktiert, bei 9 Patienten (36 %) erfolgte eine Blutentnahme, bei 7 Patienten (28 %) wurde ein EKG erstellt, bei 2 Patienten (8 %) wurde ein diagnostisch-therapeutischer Versuch mit antibiotischen Augentropfen durchgeführt und ein Patient (4 %) erhielt eine Duplexsonographie der Karotiden.

Durch die zusätzliche Diagnostik der nichtophthalmologischen Fachdisziplinen kam es zu einer Verzögerung der Diagnose auf den Folgetag bei 6 Patienten sowie auf jeweils 14 Tage bei 2 Patienten. Bei den beiden letztgenannten Patienten handelte es sich um diejenige Patienten, die antibiotische Augentropfen erhielten. Langfristige Verlaufsdaten lagen uns lediglich bei einem der beiden Patienten vor. Sowohl eine funduskopisch beschriebene irreversible Sehnervenschädigung als auch ein fortgeschrittener irreversibler Gesichtsfelddefekt waren festzustellen (Follow-up: 8,3 Monate), wobei uns der Ausgangszustand vor dem akuten Winkelblock nicht vorlag. Verlaufsdaten der Patienten mit Verzögerung auf den Folgetag lagen uns bei einem der 6 Patienten vor, wobei hier keine glaukomatöse Schädigung festzustellen war (Follow-up: 2,8 Monate). Insgesamt 3 Patienten wurden zunächst vor Konsultation eines Ophthalmologen stationär in einer anderen Fachdisziplin aufgrund der Symptome des akuten primären Winkelblocks aufgenommen.

## Diskussion

Insgesamt zeigte sich in unserer retrospektiven Auswertung mit 56 % eine hohe Rate an zusätzlich durchgeführter Diagnostik vor der Augeninnendruckmessung, wenn sich Patienten mit akutem primärem Winkelblock initial in einem nichtophthalmologischen Fachbereich vorstellten. Auf alle eingeschlossenen Patienten unabhängig vom Vorstellungsverhalten übertragen bedeutet dies, dass bei 15 % der Patienten nichtzielführende diagnostische Mittel irrtümlich indiziert wurden und insbesondere bei 9 % eine kraniale Bildgebung und bei 2 % eine Lumbalpunktion erfolgte.

Dabei ist vor allem die hohe Rate an Bildgebungen und Liquorpunktionen aus gesundheitlicher, aber auch ökonomischer Sicht relevant. Die mittlere effektive Strahlendosis einer CT des Schädels wird mit 2,1 mSv angegeben [15], wobei dieser Untersuchung gemäß Strahlenschutzgesetz eine sog. rechtfertigende Indikation voraus gehen sollte. In unserer Auswertung wurden 7 Patienten dieser unnötigen Strahlung ausgesetzt. Bei der durchgeführten Schädel-MRT liegt zwar keine Strahlenbelastung vor, jedoch handelt es sich um eine zeit- und kostenintensivere Untersuchung. Hingegen waren die beiden Patienten, bei denen eine diagnostische Lumbalpunktion durchgeführt wurde, vermeidbaren Risiken dieser invasiven Prozedur ausgesetzt, wobei die zerebrale Herniation, die bakterielle Meningitis und die zerebrale Sinusvenenthrombose zu den schwerwiegenden, wenn auch seltenen Komplikationen zählen [4]. In einer retrospektiven Studie aus Israel von Nesher et al., die 30 Patienten mit Kopfschmerzen aufgrund eines subakuten Winkelblockglaukoms einschlossen hat, zeigte sich ebenfalls eine hohe Rate an bildgebender Diagnostik bei 43 % aller Patienten. Sie führten dies auf die hohe Prävalenz und das breite differenzialdiagnostische Spektrum von Kopfschmerzen zurück, sodass teilweise Patienten die Beschwerden nicht erwähnen, als auch auf die unzureichende Kenntnis des Winkelblockglaukoms durch die medizinische Gesellschaft [9].

Eine nichtzielführende Diagnostik kann zudem auch zu einer Verzögerung der Diagnose führen. In unserer Untersuchung kam es bei 2 Patienten zu einer deutlichen Verzögerung von jeweils 14 Tagen, wobei bei einem Patienten nach einem Follow-up von 8,3 Monate eine deutliche irreversible glaukomtypische Schädigung mit fortgeschrittener irreversibler Gesichtsfeldeinschränkung beschrieben wurde. Eine Aussage, ob es sich hierbei rein um Spätfolgen des akuten Ereignisses handelt oder bereits zuvor ein Engwinkelglaukom vorlag, kann bei fehlender Voruntersuchung nicht getroffen werden. Insgesamt ist die Datenlage bezüglich der Entwicklung eines Engwinkelglaukoms bei Kaukasiern nach akutem Winkelblock gering. Hinsichtlich der Konversionsrate in ein manifestes Glaukom bei Kaukasiern existiert nur ein Bericht von Andreatta et al., bei deren Untersuchung alle Patienten ausgeschlossen wurden, die während des akuten Ereignisses bereits an einem Glaukom litten. Bei einem mittleren Follow-up von 27 ± 14 Monaten entwickelten nach akutem Winkelblock 15 % der betroffenen Patienten ein Engwinkelglaukom, 6 % erblindeten und 10 % hatten eine Visuseinschränkung. Als Risikofaktoren der irreversiblen Sehnervenschädigung zeigten sich die Länge der Symptomdauer vor Konsultation und die benötigte Zeit zur Unterbrechung des Anfalls [1].

Der hohe Anteil von 28 % aller Patienten, die aufgrund ihrer Beschwerden bei akutem primärem Winkelblock nicht initial einen Augenarzt aufsuchen, unterstreicht ebenfalls die heterogene und unspezifische Symptomatik der Erkrankung. In unserer Analyse stellten sich Patienten mit Kopfschmerzen (*p* < 0,001), Übelkeit/Erbrechen (*p* = 0,007) und Pupillenstarre/Anisokorie (*p* < 0,001) sowie Augenrötung (*p* = 0,004) signifikant häufiger beim Nichtophthalmologen vor. Einen signifikanten Unterschied bezüglich der Symptome und der Entscheidung zur zusätzlichen Diagnostik durch den Nichtophthalmologen zeigte sich nicht. In der Literatur wird beschrieben, dass die Mehrheit der Patienten, die einen akuten Winkelblock erlitten haben, nicht von ihrem Risiko bei engem Kammerwinkel wussten [8]. Zudem stellt der akute primäre Winkelblock eine eher seltene Erkrankung bei Kaukasiern dar. Die Inzidenz wird mit 2,2–4,1 Fällen pro 100.000 Einwohnern jährlich im europäischen Raum [3, 5, 12, 16] angegeben, wohingegen sie in Singapur mit 12,2 Fällen jährlich je 100.000 Einwohner über 30 Jahren weitaus höher ist [14]. Jedoch können auch andere Erkrankungen und Glaukomformen zu einem starken Augeninnendruckanstieg mit massiven Symptomen und Gefahr der Sehnervenschädigung bei verzögerter Diagnose und Therapie führen. Deshalb sollte bei den geschilderten Symptomen auch stets an die Möglichkeit eines akuten Augeninnendruckanstiegs gedacht und eine palpatorische Augeninnendruckmessung auch durch einen Nichtophthalmologen erwogen werden.

## Fazit für die Praxis

- Die Kenntnis der Symptome eines akuten Winkelblockes sind in nichtophthalmologischen Fachbereichen aufgrund der dort häufig beobachteten Erstkonsultation hochrelevant.
- Die palpatorische Augeninnendruckmessung stellt eine wichtige Untersuchungstechnik zur schnellen Eingrenzung der Differenzialdiagnosen dar.
- Entsprechende Schulungen können darauf hinwirken, dass bei unspezifischen Symptomen, wie Kopfschmerzen, Übelkeit und Erbrechen, sowie bei Pupillenstarre auch an die Differenzialdiagnose eines akuten Augeninnendruckanstiegs gedacht wird.
- Dies kann dazu beitragen, dass die hier beobachtet hohe Rate an nichtzielführender Diagnostik und die zeitliche Verzögerung mit Gefahr der irreversiblen Sehnervenschädigung reduziert werden.
